# Accelerated Discovery of the Polymer Blends for Cartilage Repair through Data-Mining Tools and Machine-Learning Algorithm

**DOI:** 10.3390/polym14091802

**Published:** 2022-04-28

**Authors:** Anusha Mairpady, Abdel-Hamid I. Mourad, Mohammad Sayem Mozumder

**Affiliations:** 1 Chemical and Petroleum Engineering Department, UAE University, Al Ain P.O. Box 15551, United Arab Emirates; mairpadi9@gmail.com; 2 Mechanical and Aerospace Engineering Department, UAE University, Al Ain P.O. Box 15551, United Arab Emirates; ahmourad@uaeu.ac.ae; 3 National Water and Energy Center, United Arab Emirates University, Al Ain P.O. Box 15551, United Arab Emirates

**Keywords:** machine learning, multinomial logistic regression, data mining, articular cartilages, mechanical properties, polymer informatics

## Abstract

In designing successful cartilage substitutes, the selection of scaffold materials plays a central role, among several other important factors. In an empirical approach, the selection of the most appropriate polymer(s) for cartilage repair is an expensive and time-consuming affair, as traditionally it requires numerous trials. Moreover, it is humanly impossible to go through the huge library of literature available on the potential polymer(s) and to correlate the physical, mechanical, and biological properties that might be suitable for cartilage tissue engineering. Hence, the objective of this study is to implement an inverse design approach to predict the best polymer(s)/blend(s) for cartilage repair by using a machine-learning algorithm (i.e., multinomial logistic regression (MNLR)). Initially, a systematic bibliometric analysis on cartilage repair has been performed by using the bibliometrix package in the R program. Then, the database was created by extracting the mechanical properties of the most frequently used polymers/blends from the PoLyInfo library by using data-mining tools. Then, an MNLR algorithm was run by using the mechanical properties of the polymers, which are similar to the cartilages, as the input and the polymer(s)/blends as the predicted output. The MNLR algorithm used in this study predicts polyethylene/polyethylene-graftpoly(maleic anhydride) blend as the best candidate for cartilage repair.

## 1. Introduction

Cartilages are the connective tissues mostly present in the long bones in the human body. Their primary functions are to provide lubrication and to act as a cushion against the friction on movement. The damage to these tissues can occur due to trauma, obesity, aging, osteoarthritis, and by several other factors. Often, even a minute tear in the cartilage over time leads to further irreversible damage [1,2]. Patients with the disintegration of cartilages experience debilitating joint pain followed by restricted movement [3,4]. Alarmingly, more than 200 million people are suffering from osteoarthritis daily around the globe [5]. 

Chondrocytes play a significant role by producing the extracellular matrix (ECM) sought for the repair of cartilages. However, the chondrocytes have only a limited capacity for self-renewal; this makes the cartilage repair difficult [6,7]. Therefore, the insertion of cartilage substitutes is deemed to be the potential solution. The damaged cartilages are often replaced by using several surgical procedures such as total knee replacement, microfracture, and mosaicplasty. Moreover, as a possible therapeutic option, the chondrocytes extracted from the donors are transplanted to the damaged area to reduce the severity of the disease [8]. However, the rejection of the implanted chondrocytes by the recipient(s) makes this procedure unpredictable, and finding the right donor is also troublesome. Especially in the case of chondrocytes, instability of the monolayer is a crucial obstacle [9,10]. Therefore, it is evident that none of these techniques offers long-lasting solutions to cartilage damage-related diseases [11,12].

Recently, tissue engineering has delivered promising results in the field of cartilage regeneration and repair. The 3D scaffolds play a crucial role in replacing biological tissues through the development of fully functioning load-bearing biomaterials [13,14]. Fabricated 3D scaffolds should have the capacity to considerably mimic the characteristics and functions of the extracellular matrix (ECM) of the cartilages [15]. The 3D scaffolds should acquire mechanical integrity and appropriate cell attachment, cell adhesion, and cell proliferation. Over the years, the polymers have shown tremendous potential to be molded as 3D scaffolds having the abovementioned properties [16,17]. By using many different combinations of natural and synthetic polymers, many have attempted to develop fully functioning and weight-bearing cartilages. However, the complexity of natural cartilages makes it very challenging to create the designed biological substitutes. As a whole, the advanced biomaterials typically fall short either in biomechanics or in functioning [18,19]. 

Moreover, the discovery of novel combinatory materials for cartilage tissue engineering typically takes a long time (i.e., 10–20 years) from the material design to commercialization. In particular, the material design procedure is one of the most tedious, time-consuming, and costly affairs in this regard [20,21]. Because going through material design and development is such a lengthy procedure, most of the time the developed product turns out to either be outdated, or the initial hypothesis of the researchers becomes irrelevant or inadequate due to the advancement of research in the respective field. Moreover, during the period of evolution of designing a commercial product, an immense amount of data is being generated in the relevant field(s). Manually, it becomes laborious and time-consuming to find and interpret the data patterns or to extract any meaningful information out of them. With the advancements in information technology, information can be retrieved from these data to implement knowledge discovery by data mining through machine-learning algorithms [22,23,24]. The process and tools of data mining provide immense help in executing the algorithms needed for material informatics [25,26]. In material informatics, a vast amount of data in the form of experimental outcomes from the previous research is being retrieved, and using the tools of machine learning, facilitation of knowledge discovery is implemented [23]. Indeed, the fourth dimension of material science involves extracting information from the literature with the aid of machine-learning algorithms. In this way, the knowledge can be retrieved by discovering the association between the data, pattern recognition, and clustering without any human intervention. This approach leads to speedy design and development of novel materials, as once the information is attained, a minimal amount of trial and error is needed to be carried out [27]. 

Consequently, the use of material informatics in developing new materials from the potential polymer(s) is currently in great demand. More precisely, in recent years, the implementation of material informatics and machine learning in materials science (i.e., polymer design, feature selection) has increased exponentially [26,28,29]. In material science, experimental design can be carried out through direct design and inverse design approaches. A direct or conventional design approach involves the prediction of the properties of the fabricated materials by taking “materials” as the input. Recently, with the advancement of machine learning, a new technique of material design, namely inverse design, can be implemented. Inverse design is a fully data-driven approach that predicts the target materials by putting the relevant material properties (i.e., molecular structures, physical, mechanical, thermal, biological, etc.) as the input [30,31]. 

For example, Venkatraman et al. (2018) used an evolutionary algorithm for the virtual screening of several classes of monomers while developing a batch of polymeric materials with a high refractive index to determine which chemical groups have a major effect on increasing the refractive indices of the developed materials [32]. Similarly, Tao et al. (2021) carried out a comparative study on the capability of 79 different machine-learning algorithms to predict the glass transition temperature of polymers. The random forest was found to be ideal in the prediction of glass transition temperature by using a large database of polymers as input [33]. Also, the heat capacity of the polymer was predicted with good accuracy by using an artificial neural network by Ishikiriyama (2021). By using the data found in ATHAS data bank artificial neural network could predict the heat capacity with minimum error [34]. Very recently, Chen et al. (2021) synthesized a hand-crafted new polymer using machine-learning techniques. This study involves the creation of a polymerization database comprised of information regarding the reactants, homopolymers, and the polymerization paths that were used to predict the synthesis pathway of the new polymer comprising of the targeted properties [35]. In another study, Le (2020) used the Gaussian process regression method to predict the tensile strength of the nanocomposites by setting the types and mechanical properties of the polymer matrices, types, and properties of carbon nanotubes as nanofillers and incorporation parameters as inputs [36]. While Venkatraman et al. (2018) [32] and Le (2020) [36] adopted a direct design approach, in a recent study, Kim et al. [30] developed a deep-learning neural network inverse design model to predict high-performance organic molecules by creating a relationship between the structure and their material properties. Very recently in 2020, Kim et al. [37] employed the inverse design approach through a neural network algorithm in which 31,713 known zeolites properties were considered as input to predict 121 porous nanostructures. 

To the best of the authors’ knowledge, no study has yet been conducted to predict the polymer(s)/blend(s) to mimic human cartilages by a machine-learning algorithm. The primary objective of this study is to implement an inverse design approach to obtain the target polymer(s)/blend(s) that exhibit similar properties of the human cartilage. In this study, the null hypothesis assumes that the prediction of polymer/blends’ names in the database and the subset is quite similar. However, the alternative hypothesis would be that the prediction between the sets is dissimilar in nature. This research was carried out in four steps; initially, the systematic bibliometric analysis was carried out by using the review articles’ citation data in the field of cartilage tissue engineering, and then the relevant database was created from the PoLyInfo library by using data-mining tools. Then a machine-learning technique (i.e., multinomial logistic regression) has been used to run both single and multiple properties optimizations. In the final step, the machine-learning algorithm was employed to predict the polymer(s)/blends that possess similar functional properties of the human cartilages (e.g., tensile modulus, tensile strength, and elongation at break). 

## 2. Methodology

### 2.1. Bibliometric Analysis

Bibliometric analysis is a powerful tool that allows researchers to get an overview of the trend in which the specific research field is heading into. The benefit of this analysis includes the extraction of the original articles and their citation summary to run the overall publication analysis in a particular field of interest [38,39]. From the large group of polymers and subgroups of polymers available in the market, the objective of this study was to discover the polymers/composites which are among the best to be used in cartilage repair. To retrieve the major groups of polymers/composites, a bibliometric analysis was carried out. In this study, using “cartilage” as the keyword, review journal articles’ title, abstracts and their citation reports were extracted, and bibliometric analysis was run in R program. The results containing the top ten highly cited articles were tabulated and summarized in Table 1. Each review paper linked to cartilage repair was manually reviewed, and the names of the major polymers/composites mentioned in these papers were extracted and listed. The selection of these polymers/composites was done based on their recurrent usage in cartilage tissue engineering. The final selection of the polymers/blends was made based on the availability of data in the Polyinfo database on January 2021 summarized in Table 2.

### 2.2. Database Creation

For the success and durability of the biomaterials, mechanical properties play a substantial role [50,51]. Specifically, in cartilages, a primary symptom of the disease (i.e., osteoarthritis) is the deterioration of the mechanical properties of the cartilages [52]. Concerning the biomechanical properties of the cartilages, the tensile strength, tensile modulus, and elongation at break are the most sought mechanical properties, because the main function of cartilage is to hold/resist the amount of stress and compressive force exerted on the body part(s) of interest at any given moment [53]. The key mechanical properties of the native articular cartilages were extracted from the literature by using data-mining tools and are summarized in Table 3. The tensile strength, tensile modulus, and elongation of the natural cartilages reported in Table 3 are 35 MPa, 3–100 MPa, and 2–140%, respectively However, under 15% less strain, the tensile modulus reaches only up to 5 to 10 MPa [54]. Therefore, the database of the polymers/composites has been created taking into account these key mechanical properties of the natural cartilages.

**Figure 1:**
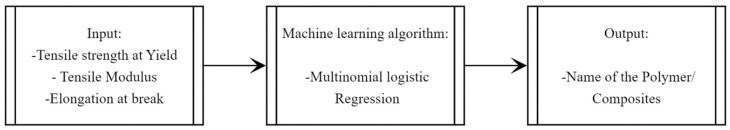
Inverse design approach.

PolyInfo is a section of the NIIMS materials database that extracts numerical data from the relevant sources (i.e., academic articles) [58]. In this study, data-mining tools were used to retrieve the numerical values of the major mechanical properties of the polymers/blends used in cartilage repair from the PolyInfo database. The summarized database (Table 2) includes a collection of 97 polymers/blends and their related mechanical properties. The ranges of the extracted values for each of the mechanical properties were chosen as the input or independent variable in this study, whereas the names of the polymers/blends were taken as the output or the dependent variable (i.e., categorical in nature) for the machine-learning algorithm. The input and output variables were chosen in such a way to implement the inverse design approach shown in Figure 1. Through this design approach, the polymers/blends’ names were predicted by using properties of the natural cartilage extracted from journal articles (summarized in Table 3).

### 2.3. Multinomial Logistic Regression (MNLR)

For dealing with the categorical dependent variable with multiple levels, very few modeling techniques are available. Among those few techniques, multinomial logistic regression (MNLR) is one of the most suitable machine-learning algorithms used to model the data having multiple factors and levels. The dataset used to implement the multinomial logistic regression technique is typically categorical and has multiple levels. This approach can deduce the probability of occurrence of the output in the dataset. This regression is distinct from its linear regression as it implements a sigmoidal behavior to its data [59]. 

In multinomial logistic regression, the model calculates the probability of the one factor chosen in place of the other. The probability mass function is given by Equation (1) [60]:

(1) $$Pr\;\left(n_{1},\ldots \ldots ..,n_{k}\right)=\frac{n!}{n_{1}!n_{2}!\ldots \ldots n_{k}!}p_{1}^{n_{1}}p_{2}^{n_{2}}\ldots \ldots \ldots \ldots ..p_{k}^{n_{k}}.$$

Equation (2) can be used to calculate the log-likelihood function:

(2) $$L\left(\beta \right)=\sum_{i=1}^{k}\left\{y\left(x^{\prime }\beta_{1}\right)+\ldots y\left(x^{\prime }\beta_{k}\right)-ln\left(1+exp\left(x^{\prime }\beta_{1}\right)\ldots +exp\left(x^{\prime }\beta_{k}\right)\right)\right\}\;$$

wherein the $\left(x^{\prime }\beta_{j}\right)$ can be computed by using the Equation (3)

(3) $$\left(x^{\prime }\beta_{j}\right)=ln\left(Pr\;\left(y=\frac{j}{x}\right)\right)/\left(Pr\;\left(y=\frac{1}{x}\right)\right),\;j=1,2,\ldots \ldots ..k.$$

To evaluate the modeled data having a categorical response variable, it is crucial to develop a relationship between the logarithm odds and the explanatory variables for the modeled data. It is given by Equation (4):

(4) $$\log\left(\frac{p}{1-p}\right)=\beta_{0}+\beta_{1}x_{1}+\ldots +\beta_{i}x_{i}$$

where *x* is the explanatory variable, *βs* are the regression coefficient of the factor(s), and *p* is the predicted probability. In dealing with the multiclass regression problem, a relationship between the input and output is developed by Equation (5):

(5) $$P\left(y=k/x\right)=\frac{exp^{\left(\beta_{0}^{k}+\beta_{1}^{k}x_{1}+\ldots \ldots \ldots +\beta_{i}^{k}x_{i}\right)}}{\sum_{J=1}^{K}exp^{\left(\beta_{0}^{k}+\beta_{1}^{k}x_{1}+\ldots \ldots \ldots +\beta_{i}^{k}x_{i}\right)}}$$

where *k* is the number of classes and *βs* are the regression coefficient of the factor(s) [61]. 

**Figure 2:**
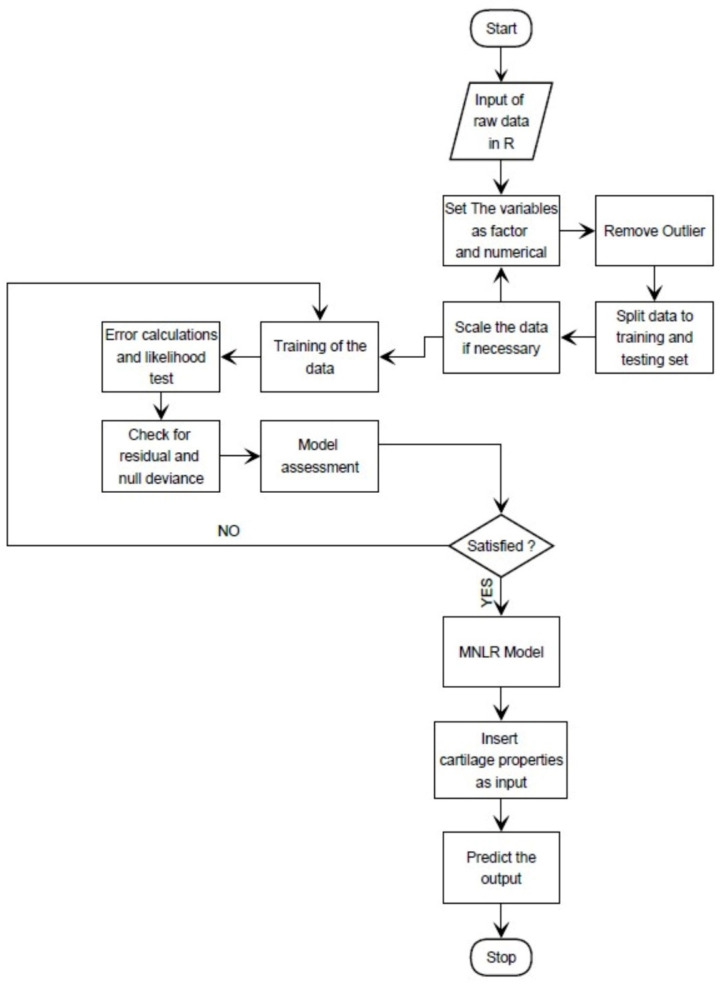
Flowchart of the operation of the multinomial logistic regression (MNLR).

The overall workflow of MNLR is depicted in the form of a flowchart in Figure 2. The initial step includes the preprocessing of the data, as the computer cannot differentiate between the factorial and numerical variables. Therefore, each parameter was needed to be assigned as either numerical or categorical. The final step of the data preprocessing includes the removal of the outlier(s) from the dataset. To check the accuracy of the prediction, the data were divided into training and testing sets. Then the training set was being fed into the algorithm and the likelihood ratio test was performed. The deviance of the null hypothesis and the residual was noted. The model’s goodness of fit was confirmed. By using the testing data without the output, a new prediction was retrieved. Once the difference between the observed and the predicted values (i.e., residual) was minimum, the prediction was done by using the tensile modulus, tensile strength, and the elongation at break of the natural cartilages.

## 3. Results and Discussion

### 3.1. Bibliometric Analysis

The most convenient and least time-consuming approach to obtain an overall standing (i.e., trend, current progress, etc.) of any research field is the bibliometric analysis. It enables the researchers to summarize the overall research trends and to develop the link between the variables in the field(s). The bibliometric analysis can be used to analyze the most evaluated component(s) in the area of the research [62,63]. Particularly in tissue engineering, a huge number of materials/blends/composites are being investigated to evaluate their efficacy to replace damaged or degrading cartilages. Among them, polymers are at the frontline in creating biomaterial substitutes (i.e., scaffolds) [64,65]. In cartilage tissue engineering, several different types and combinations of polymers are being investigated to mimic articular cartilages [8,66]. To select the most suitable polymer(s) and/or the combination of polymers, the bibliometric analysis was used in this study. The review papers were extracted from the Web of Science by using the “cartilages” and “polymers” as the keywords. The review articles’ citation details were downloaded for the period from 2005–2020. By using the bibliometrix package in R program [67], a list of highly cited review papers was extracted and the top ten cited papers are being summarized in Table 1.

**Figure 3:**
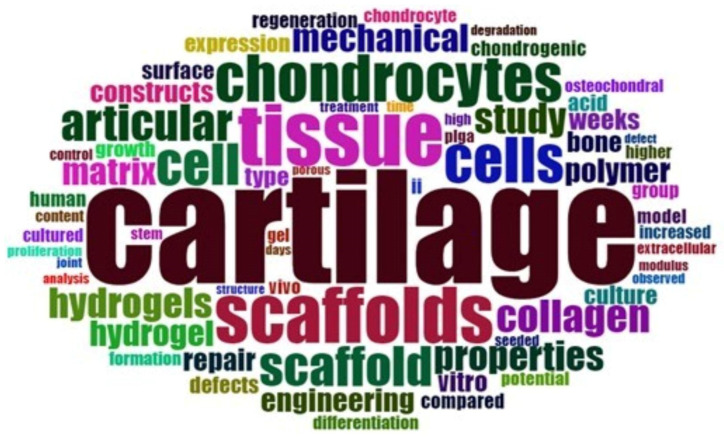
Wordcloud of the most recurrently (>70 times) occurred words found in the articles’ abstracts using “polymers” and “cartilages” as the keywords.

Upon running the bibliometric analysis using “cartilages” and “polymers” as keywords, the most recurrent words were displayed in the form of the wordcloud as shown in Figure 3. All of the keywords shown in the wordcloud appeared more than 70 times in the published literature. In Figure 3, the keywords are displayed in larger to smaller fonts depending on their recurrence in the literature. It is evident from Figure 3 that cartilage, scaffolds properties, collagen, polymers, hydrogels, mechanical strengths, and chondrocytes are found to be among the most recurrent keywords. In other words, these are the most important parameters to consider while designing a new material for cartilages repair. In this study, our focus was limited to the mechanical strength of the polymer(s) to mimic the articular cartilages. Considering the mechanical properties (i.e., tensile strength, tensile modulus, and elongation, etc.), based on the recurrent mentions in the review papers and the data available in the PoLyInfo database, the list of polymers/blends has been prepared to be used in the machine learning algorithm (shown in Table 2).

### 3.2. Selection and Preprocessing of the Database

Depending on the load or the direction of stretching, the components of cartilages, especially the collagen fibrils and proteoglycans, move towards the direction of the load. Initially, when the tensile stress is less, only the collagen fibers’ realignment occurs [68]. Once the cartilage experiences large deformation, the collagen attains a large amount of tensile stiffness due to the stretching of collagen fibers. Once the tension is removed, the collagen fibrils and proteoglycans move back to their normal position. Indeed, the viscoelasticity of cartilages in tension is best described by the mechanical properties, such as tensile strength, elongation at break, and tensile modulus [68,69]. Therefore, in this study, the ranges of the tensile strength, elongation at break, and tensile modulus have been considered for the database to take account of the viscoelastic behavior of cartilages.

**Figure 4:**
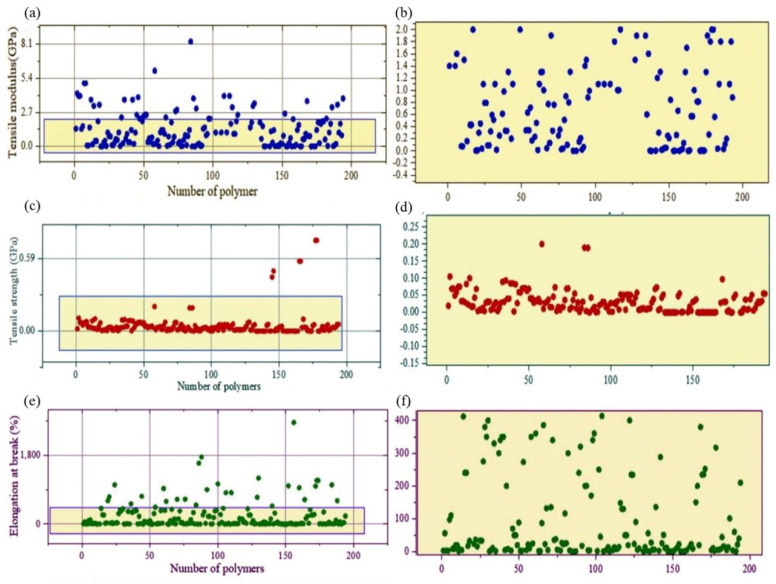
Scatter plots. (**a**) Overall data of the tensile modulus of the polymer(s)/blend(s) in the database. (**b**) Zoomed image of the highly-dense region of the tensile modulus data. (**c**) Overall data of the tensile strength of the polymer(s)/blend(s) in the database. (**d**) Zoomed image of the highly dense region of the tensile strength data. (**e**) Overall data of the elongation at break (%) of the polymer(s)/blend(s) in the database. (**f**) Zoomed image of the highly dense region of the elongation at break data.

Typically, in the inverse design approach, the properties of the polymers/blends are used as the input whereas the output is the most suitable blend to be used in the intended applications. In this study, the input is the numerical range of the selected properties, and the output is found as a categorical variable (i.e., string or the text). For this purpose, the scattered plots have been plotted in Figure 4 to represent the raw data for tensile modulus (Figure 4a,b), tensile strength (Figure 4c,d), and elongation at break (Figure 4e,f), respectively. It is important to note that the database has been created based on the list of the polymers/blends most recurrently used in cartilages repair. The raw data retrieved from the databases consisted of outliers that were needed to be screened/removed before running the machine-learning algorithm. After cleaning the outliers, the most concentrated data zones were selected for all three properties of interest. As shown in Figure 4, the tensile strength data is so concentrated that they almost formed a straight line, whereas the tensile modulus and elongation data shown in Figure 4 were a little more scattered. The blue rectangular boxes shown in each of Figure 4a–f represent the numerical ranges of tensile modulus, tensile strength, and elongation, respectively. Upon cleaning up the outliers, the magnitude range of the tensile modulus, tensile strength, and elongation was found to be 0–2 GPa, 0–0.2 GPa, and 0–400%, respectively. These ranges are in agreement with the mechanical properties of human cartilages presented in Table 3.

### 3.3. Multinomial Logistic Regression (MNLR)

**Figure 5:**
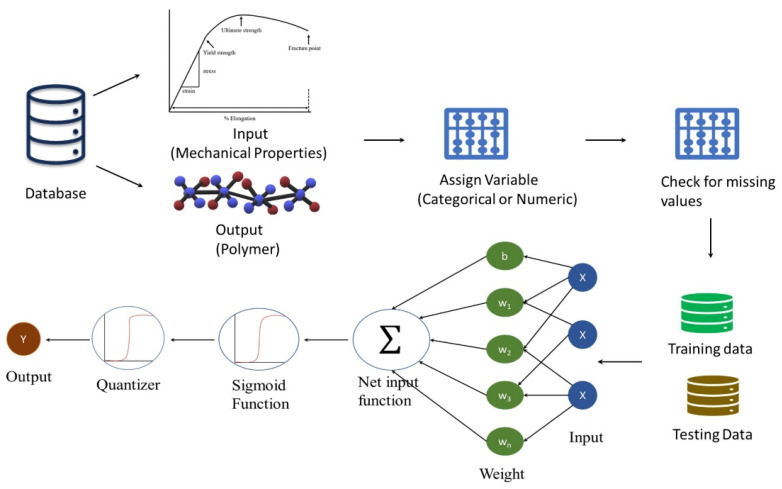
Schematic representation of data modelled by multinomial logistic regression.

In this study, numerical independent variables (i.e., inputs) and categorical response variables (i.e., the outputs) were used. Indeed, the response variables were 97 different polymers/blends, and consist of multiple levels; hence, the multinomial logistic regression (MNLR) was deemed to be suitable for modeling the response variables as factors [59,70,71]. The numerical factors were at two levels, and they consisted of a range of minimum and maximum values of the tensile strength at yield, tensile modulus, and elongation at break. The input was either an individual factor or a combination of multiple factors for multivariable optimization. Figure 5 shows the schematic diagram of the whole simulation process. Once the data is divided into 75% as training data and 25% as testing data, the formula and vectors in which data is assigned are needed to run the machine-learning algorithm. Once the algorithm’s simulation is completed, the values such as the goodness of fit, likelihood ratio, and its capacity to reject the null hypothesis is reviewed. Once the model efficacy is verified, the user-defined inputs are inserted into the algorithm to predict the output. For example, taking the tensile modulus of blends as the input (i.e., single factor), the training datasets are modeled. After modeling with the training data, the range of the tensile modulus of the cartilages was used as the testing input to predict the best polymer blends owing to having similar properties of the cartilages. The response variables were found to be the blends of poly(glycolic acid)//poly(lactic acid) and poly(methyl methacrylate)//poly(epsilon-caprolactone).

The goodness-of-fit model was assessed by comparing its residual deviance (D_m_ = −2 LL_m_ = 1466.6345) with the null hypothesis residual deviance for the model (D_0_ = −2 LL_0_ = 1763.898), which includes only the intercepts. The deviance is a measure of how poorly the model reproduces the observed data. The likelihood ratio test (G = D_0_ − D_1_ = 297.26296, df = 94, *p* < 0.001) compares these two deviances. The null hypothesis is rejected, indicating a statistically significant decrease in deviance when the predictor (X) is included in the model. This means that the model fits the data better than the null model in terms of the correspondence between the observed and predicted conditional probabilities. The goodness-of-fit of modeled data was interpreted by utilizing *p*-value, and the residual deviation and its corresponding *p*-value were summarized in Table 4. It is evident from Table 4 that the null hypothesis was rejected for all of the independent variables, and thereby, the *p*-value is significant for all of the parameters (*p* < 0.05).

**Figure 6:**
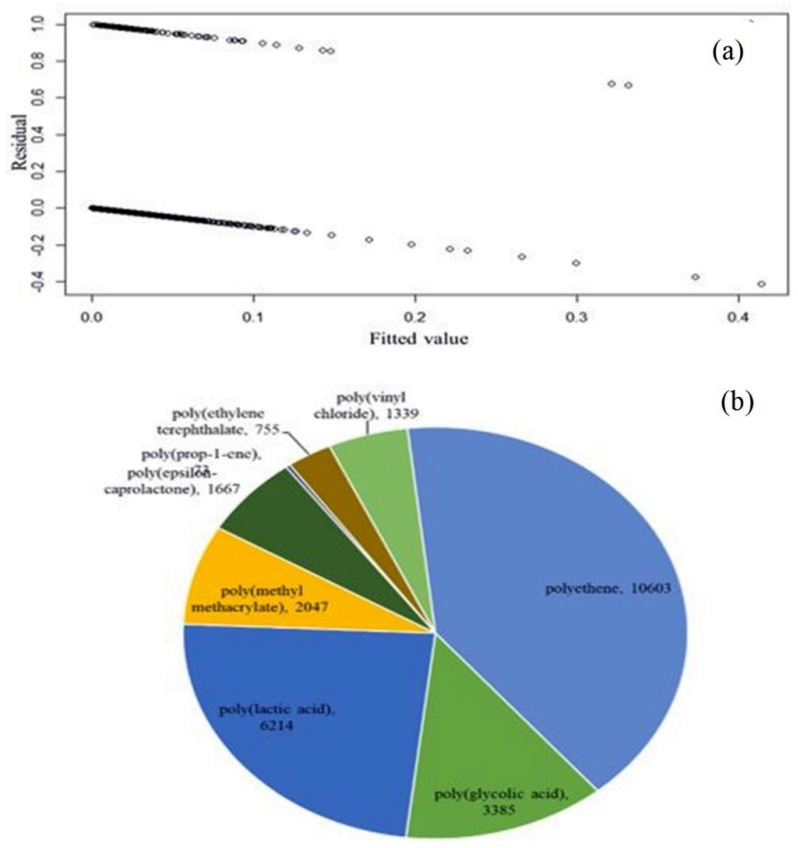
(**a**) Scatter plot of the residual verse fitted value. (**b**) Pie chart displaying the number of times the MNLR algorithm predicted polymers/blends appeared in the ScienceDirect and PubMed journals.

The MNLR model was run by using the neural network pack in R after 100 iterations [72]. The residual values have been plotted against the fitted values to generate the scatter plot (Figure 6a) while considering all three independent variables (i.e., tensile modulus, tensile strength, and elongation at break of the natural cartilages) used in this study for multivariable optimization. The scatter plot presented in Figure 6a proves the data independence, homoscedasticity, and linearity. On inserting the tensile modulus of 3–100 MPa, elongation of 2–140%, and the tensile strength of 35 MPa to the already fitted model, the multinomial regression model predicted polyethene/polyethene-graft-poly(maleic anhydride) blend as the most suitable one for the cartilage repair. The predicted results along with the residual deviance for all other individual and combinatory testing inputs are summarized in Table 5.

To confirm whether the predictions made by the MNLR model are accurate and relevant to cartilage tissue engineering, the predicted polymers/blends’ names were chosen as the keywords in PubMed and ScienceDirect and searched. The search results were summarized in the form of a pie chart, as shown in Figure 6b. It was found that polyethylene and polylactic acid have been mentioned with cartilage tissue engineering 10,603 and 6214 times, respectively. 

Rise in the use of the polycaprolactone (PCL) in the field of cartilage tissue engineering is attributed to the minimal intermolecular interaction and high movement in the chain segment [73,74]. This mobility facilitates the design the PCL scaffolds in the form of composites, foams and fibers [75,76]. The natural polymers like chitosan and collagen with PCL is proven to improve the crosslinking as well as its mechanical properties. Moreover, the application of the crosslinking agents to hydrophobic polymers is known to improve their water uptake and convert them to hydrophilic polymers [77,78]. The mechanical properties of PCL are highly influenced by its molecular weight. For instance, the scaffold made up of PCL having the molecular weight of 15,000 g/mol is known to exhibit brittle characteristics, whereas the scaffold consisting of 40,000 g/mol is soft and semicrystalline in nature [73,74]. A second attractive feature of PCL and polyethylene is their biodegradability, and they dissolve conveniently in presence of enzyme activity and follows a natural metabolic pathway. PCL is also used as crosslinking agents in many studies as they blend with most of the polymers easily [73,74]. 

Similarly, the mechanical properties of polyethylene are highly influenced by the molecular weight. Best wear performance is observed with the polyethylene consisting of 1 million repeating units. The high-density polyethylene is known to have a lesser degree of branching, which is attributed to its recommendable intermolecular forces and tensile strength. Along with the molecular weight, crosslinking agents and crystallinity also impact their mechanical properties. Normally polymers having lower crystallization temperature contain numerous amorphous regions which weaken the overall mechanical strength of the materials. However, the addition of nanofillers, acting as nucleation agents, to polyethylene is known to increase its crystallinity and mechanical properties [79,80].

In addition, the mechanical properties of polymers or blends are influenced by several factors, such as the molecular weight, degree of polymerization, and the cross-linking agent. The influences vary from one polymer to another. For example, as mentioned earlier, the increase in molecular weight in PCL (above 40,000 g/mol) improves its mechanical properties. However, in case of poly(ethylene glycol) diacrylate, the rise in molecular weight of polymer blend leads to its improved mechanical properties, but has negative impact on the cell growth [81]. The crystallinity of the polymer also contributes to the mechanical properties of the polymers. The mechanical strength increases with a rise in crystallinity. However, the studies indicate that the cell attaches more in the amorphous region than in the crystalline region, which is due to the surface roughness as the amorphous regions are rougher than the crystalline regions [80]. More specifically, chondrocytes are known to attach at higher concentration at PGA than in PCL [80].

Moreover, polylactic acid and polycaprolactone belong to the group of linear aliphatic polyester polymers [82] and polycaprolactone is known to increase cell viability by 20% [83]. Even the byproducts of the degradation of polylactic acid (i.e., water and carbon dioxide) are non-toxic in nature [84]. Moreover, both polypropylene and polyethylene are widely used in developing implants, as they are easy to be molded to the desired shape and are inexpensive [85,86,87,88]. They have been known to initiate a minimal immune response, and have superior mechanical (i.e., viscoelastic) properties and biocompatibility [89,90,91]. Particularly, both PLA and PCL can be modified to exhibit viscoelastic properties required for mimicking cartilages [92,93,94,95,96]. Moreover, polypropylene has proven to be an excellent candidate in the development of cartilages in nasal reconstructive surgery [97]. Overall, all the polymers/blends mentioned in the pie chart (Figure 6b) have been employed in the field of cartilage tissue engineering [89,90,98,99].

### 3.4. Conclusions

The design of new biomaterials is a complex, tedious, and time-consuming affair. Designing cartilage substitutes is even more intricate due to their unique properties/functionality and their diverse locations in the human body. Among many, viscoelasticity is one of the most important parameters that needs to be taken into serious consideration in designing cartilages. More importantly, the viscoelasticity of the cartilages may not be attributed to any single property; rather it is better represented by a set of mechanical properties such as tensile strength, tensile modulus, and the elongation at break. Therefore, it is expected that the best polymer matrices/blends to be used in cartilage repair must exhibit these properties as much as in the ranges of the properties of the natural articular cartilages. This study attempts to use the inverse design approach by using a machine-learning algorithm (i.e., multinomial logistic regression) to predict the most suitable polymers/blends for cartilage substitutes by using the ranges of the tensile modulus, elongation at break, and tensile strength of the natural cartilages as inputs. Both single and multivariable optimization was conducted so that the output was predicted by using both individual and combinatory properties of the cartilages. Considering all three properties of interest, poly(epsilon-caprolactone)/poly(bisphenol A carbonate) and polyethene//polyethene-graft-poly(maleic anhydride) were found to be the best polymer(s)/blends for cartilage repair using the multinomial logistic regression techniques. All of the predicted polymer(s)/blend(s) through this machine-learning algorithm are FDA-approved to be used in cartilage tissue engineering; more importantly, they possess the similar tensile biomechanical properties of the natural cartilages, and may only initiate minimal immune responses in the body environment.

However, the limitation of this study lies in the low level of goodness of fit of the modeled data, which is largely attributed to the response variable to be categorical in nature. The different machine-learning algorithms may be explored to handle the categorical variable(s) with multiple levels. Moreover, the biological properties of the natural cartilages may be included as inputs in future research, although there is still a lack of an appropriate database to correlate the properties of the stem cells linked to the polymer matrix/blends to be used in cartilage repair. Hence, it is crucial to encourage researchers to report the biological data to the journals in a uniform format, which will eventually help to create the database. As a result, the data mining and machine-learning approaches can be employed to predict the list of suitable polymers and/or to predict their properties to be used in several different tissue engineering applications.

## Figures and Tables

**Table 1:** Top 10 cited articles from web of science having cartilage as the keyword from Web of Science published in the years 2005–2020.

S. No.	Refs.	Publication Year	Citation	Average Citation/Year
1	[40]	2007	759	54.21
2	[41]	2009	728	60.67
3	[42]	2010	542	49.27
4	[43]	2012	447	49.67
5	[44]	2008	447	34.38
6	[45]	2007	439	31.36
7	[46]	2015	395	65.83
8	[47]	2017	361	90.25
9	[48]	2009	285	23.75
10	[49]	2011	283	28.30

**Table 2:** Overall database of polymers/blends used in cartilage tissue engineering from PoLyInfo database.

Polymers/Composites	TM (GPa)	TS (GPa)	E (%)
polystyrene	1.4	0.01913	3.5
polystyrene	4.2	0.1048	56.48
poly(2-methylstyrene)	4	0.0691	3
poly(2-methylstyrene)	4	0.0691	3
poly(vinyl alcohol)	1.4	0.049	97
poly(vinyl alcohol)	1.6	0.059	110
Polyacrylonitrile	5	0.075	8.5
Polyacrylonitrile	5	0.075	8.5
poly(N-vinylpyrrolidone)	0.075	0.0332	20
poly(N-vinylpyrrolidone)	0.075	0.0332	20
poly(methyl methacrylate)	1.5	0.02958	2.1
poly(methyl methacrylate)	3.7	0.083	6.4
poly(vinyl chloride)	0.16	0.02393	4.9
poly(vinyl chloride)	3.2	0.1	412.5
poly(1-chloro-2-hexylvinylene)	0.43	0.017	240
poly(1-chloro-2-hexylvinylene)	0.43	0.017	240
polyethene//polystyrene	2	0.0431	17.5
polyethene//polystyrene	3.3	0.0683	32.7
polyethene//poly[ethene-co-(vinyl acetate)]	0.0052	0.0044	610
polyethene//poly[ethene-co-(vinyl acetate)]	0.0076	0.0076	710
polyethene//poly(acrylonitrile-co-butadiene)	0.3	0.007	24
polyethene//poly(acrylonitrile-co-butadiene)	0.45	0.0135	37
poly[ethene-co-(oct-1-ene)]//poly[ethene-co-(vinyl alcohol)]	0.035	0.004	16
poly[ethene-co-(oct-1-ene)]//poly[ethene-co-(vinyl alcohol)]	1.1	0.029	1025
poly[(maleic anhydride)-co-styrene]//poly[ethene-co-(methyl acrylate)-co-(glycidyl methacrylate)]	0.79	0.0309	33.6
poly[(maleic anhydride)-co-styrene]//poly[ethene-co-(methyl acrylate)-co-(glycidyl methacrylate)]	0.79	0.0309	33.6
poly(vinyl acetate)//poly(3-hydroxybutyric acid)//poly(3-hydroxybutyric acid)	0.091	0.0075	275
poly(vinyl acetate)//poly(3-hydroxybutyric acid)//poly(3-hydroxybutyric acid)	0.091	0.0096	380
poly(lactic acid)//poly[ethene-co-(vinyl acetate)]	0.32	0.02202	350
poly(lactic acid)//poly[ethene-co-(vinyl acetate)]	0.59	0.0294	400
poly(2-ethylhexyl acrylate)//poly(vinyl chloride)	0.51	0.0042	1.6
poly(2-ethylhexyl acrylate)//poly(vinyl chloride)	1.1	0.0132	5.5
poly(hexano-6-lactam)	0.019	0.0278	10.7
poly(hexano-6-lactam)	2.3	0.089	330
poly[(hexane-1,6-diamine)-alt-(adipic acid)]	0.25	0.031	1.9
poly[(hexane-1,6-diamine)-alt-(adipic acid)]	3.7	0.0932	530
poly(11-aminoundecanoic acid)	0.61	0.0399	300
poly(11-aminoundecanoic acid)	0.98	0.04	340
poly(dodecano-12-lactam)	0.33	0.085	350
poly(dodecano-12-lactam)	0.33	0.085	350
poly(bisphenol A carbonate)	1.3	0.0028	5
poly(bisphenol A carbonate)	3.7	0.082	200
poly(propylene carbonate)	0.2	0.027	8.2
poly(propylene carbonate)	1.1	0.059	726.8
poly(epsilon-caprolactone)//poly(bisphenol A carbonate)	2.5	0.0583	3.5
poly(epsilon-caprolactone)//poly(bisphenol A carbonate)	3.9	0.0692	70
poly(hexano-6-lactam)//poly(bisphenol A carbonate)	2.2	0.0705	50
poly(hexano-6-lactam)//poly(bisphenol A carbonate)	2.2	0.0705	50
poly(lactic acid)//poly(bisphenol A carbonate)	2	0.063	20.3
poly(lactic acid)//poly(bisphenol A carbonate)	2.4	0.069	87.9
poly(ethylene-2,5-furandicarboxylate)	2.5	0.041	2.81
poly(ethylene-2,5-furandicarboxylate)	2.5	0.041	2.81
poly(5-hydroxy-3-oxavaleric acid)	0.34	0.0283	273
poly(5-hydroxy-3-oxavaleric acid)	0.63	0.0357	441
poly[(butane-1,4-diol)-alt-(succinic acid)]	0.34	0.0335	5.2
poly[(butane-1,4-diol)-alt-(succinic acid)]	0.71	0.0399	21.9
poly[(butane-1,4-diol)-alt-(terephthalic acid)]	0.17	0.03	10
poly[(butane-1,4-diol)-alt-(terephthalic acid)]	6	0.2	350
poly(epsilon-caprolactone)	0.23	0.0105	25
poly(epsilon-caprolactone)	0.46	0.0275	930
poly[(propane-1,3-diol)-alt-(terephthalic acid)]	0.013	0.03	360
poly[(propane-1,3-diol)-alt-(terephthalic acid)]	1.1	0.037	590
poly(3-hydroxybutyric acid)	1.3	0.0152	1.8
poly(3-hydroxybutyric acid)	1.3	0.0152	1.8
polyethene//poly(ethylene terephthalate)	1	0.046	87
polyethene//poly(ethylene terephthalate)	2.3	0.073	386
poly(prop-1-ene)//poly(ethylene terephthalate)//poly[ethene-co-(5-ethylidene-2-norbornene)-co-(prop-1-ene)]	0.045	0.0041	23.2
poly(prop-1-ene)//poly(ethylene terephthalate)//poly[ethene-co-(5-ethylidene-2-norbornene)-co-(prop-1-ene)]	0.77	0.016	132.1
poly(vinyl chloride)//poly(D,L-2-methyl-2-propyl-3-hydroxypropionic acid)	0.13	0.0072	4.8
poly(vinyl chloride)//poly(D,L-2-methyl-2-propyl-3-hydroxypropionic acid)	1.9	0.0146	648
poly(ethylene oxide)//poly(lactic acid)	0.33	0.015	135
poly(ethylene oxide)//poly(lactic acid)	0.76	0.036	340
poly[(butane-1,4-diol)-alt-(terephthalic acid)]//poly(hexano-6-lactam)	2.8	0.069	36
poly[(butane-1,4-diol)-alt-(terephthalic acid)]//poly(hexano-6-lactam)	2.9	0.07	38
poly(epsilon-caprolactone)//cellulose	0.25	0.0095	6.3
poly(epsilon-caprolactone)//cellulose	0.35	0.0142	650
poly(3-hydroxybutyric acid)//poly(lactic acid)	0.027	0.0361	3.5
poly(3-hydroxybutyric acid)//poly(lactic acid)	0.035	0.0552	27
poly(3-hydroxybutyric acid)//poly[(glycolic acid)-co-(lactic acid)]	0.51	0.012	23
poly(3-hydroxybutyric acid)//poly[(glycolic acid)-co-(lactic acid)]	0.9	0.029	116
poly(3-hydroxybutyric acid)//poly[(D-lactic acid)-co-(L-lactic acid)]	0.1	0.0016	26
poly(3-hydroxybutyric acid)//poly[(D-lactic acid)-co-(L-lactic acid)]	0.8	0.012	300
Polyetheretherketone	1.3	0.031	3.8
Polyetheretherketone	8.3	0.189	496
Polyethene	0.001	0.004	0.04
Polyethene	3.8	0.1887	1590
poly(prop-1-ene)	0.0034	0.0000174	1.6
poly(prop-1-ene)	3	0.043	1750
poly(but-1-ene)	0.15	0.01	240
poly(but-1-ene)	0.21	0.0164	320
poly(4-methylpent-1-ene)	0.021	0.0006	18
poly(4-methylpent-1-ene)	0.06	0.0018	900
polyethene//poly(prop-1-ene)//poly[ethene-co-(prop-1-ene)]	1.4	0.0205	200
polyethene//poly(prop-1-ene)//poly[ethene-co-(prop-1-ene)]	1.5	0.024	200
polyethene//polystyrene//polystyrene-block-(hydrogenated polybutadiene)-block-polystyrene	0.88	0.0302	4
polyethene//polystyrene//polystyrene-block-(hydrogenated polybutadiene)-block-polystyrene	0.99	0.0302	4
polyethene//poly[ethene-co-(maleic anhydride)]	2.2	0.0151	170
polyethene//poly[ethene-co-(maleic anhydride)]	2.2	0.0171	340
polyethene//poly[ethylene-co-(but-1-ene)]	0.58	0.003	360
polyethene//poly[ethylene-co-(but-1-ene)]	0.78	0.027	1050
polyethene//hydrogenated poly(cyclopenta-1,3-diene)	0.050	0.0019	2
polyethene//hydrogenated poly(cyclopenta-1,3-diene)	1.1	0.0265	250
polyethene//poly(hexano-6-lactam)//polyethene-graft-poly(maleic anhydride)	0.042	0.0237	45.5
polyethene//poly(hexano-6-lactam)//polyethene-graft-poly(maleic anhydride)	0.49	0.0323	413.7
polyethene//polyethene-graft-poly(maleic anhydride)	0.92	0.008	1.1
polyethene//polyethene-graft-poly(maleic anhydride)	1.1	0.0524	821
poly(prop-1-ene)//poly(hexano-6-lactam)//poly[propylene-graft-(maleic anhydride)]	0.092	0.0383	5.3
poly(prop-1-ene)//poly(hexano-6-lactam)//poly[propylene-graft-(maleic anhydride)]	4	0.051	13.5
polyethene//polyethene-graft-poly(maleic anhydride)	0.92	0.008	1.1
polyethene//polyethene-graft-poly(maleic anhydride)	1.1	0.0524	821
poly(prop-1-ene)//poly(hexano-6-lactam)//poly[propylene-graft-(maleic anhydride)]	0.092	0.0383	5.3
poly(prop-1-ene)//poly(hexano-6-lactam)//poly[propylene-graft-(maleic anhydride)]	4	0.051	13.5
poly[ethene-co-(prop-1-ene)]//poly[propylene-graft-(maleic anhydride)]	1.8	0.0272	8.7
poly[ethene-co-(prop-1-ene)]//poly[propylene-graft-(maleic anhydride)]	3.1	0.0272	8.7
poly(hexano-6-lactam)//poly[ethene-co-(prop-1-ene)]//poly[propylene-graft-(maleic anhydride)]	1	0.044	14
poly(hexano-6-lactam)//poly[ethene-co-(prop-1-ene)]//poly[propylene-graft-(maleic anhydride)]	1	0.0577	148
poly[ethylene-co-(but-1-ene)]//cellulose acetate	2	0.0079	130
poly[ethylene-co-(but-1-ene)]//cellulose acetate	2.5	0.0079	130
cellulose//poly[ethylene-co-(but-1-ene)]	0.12	0.0117	50
cellulose//poly[ethylene-co-(but-1-ene)]	0.12	0.0117	50
poly(vinylidene fluoride)	0.16	0.00945	20
poly(vinylidene fluoride)	0.16	0.00945	400
poly(tetrafluoroethylene)	0.21	0.012	234.4
poly(tetrafluoroethylene)	25	0.012	234.4
Polychlorotrifluoroethylene	0.60	0.02882	24
Polychlorotrifluoroethylene	0.60	0.03861	90
poly(methyl methacrylate)//poly(vinyl chloride)	1.5	0.0683	6.2
poly(methyl methacrylate)//poly(vinyl chloride)	1.9	0.0732	9.8
poly(ethylene oxide)	3.2	0.006895	700
poly(ethylene oxide)	3.4	0.01034	1200
poly[(glycolic acid)-co-(lactic acid)]	0.20	0.043	0.84
poly[(glycolic acid)-co-(lactic acid)]	0.50	0.052	14
poly(glycolic acid)//poly(lactic acid)	0.0028	0	3
poly(glycolic acid)//poly(lactic acid)	1.9	0	56
poly(lactic acid)//poly[(glycolic acid)-co-(lactic acid)]	0.6	0	1.6
poly(lactic acid)//poly[(glycolic acid)-co-(lactic acid)]	1.6	0	28
poly(methyl methacrylate)//poly(epsilon-caprolactone)	0.0038	0	6.3
poly(methyl methacrylate)//poly(epsilon-caprolactone)	0.001	0	6.7
poly(vinyl chloride)//poly(epsilon-caprolactone)	0.37	0	136
poly(vinyl chloride)//poly(epsilon-caprolactone)	0.39	0	470
poly(epsilon-caprolactone)//poly(lactic acid)	0.0023	0.0472	3
poly(epsilon-caprolactone)//poly(lactic acid)	1.2	0.0554	288
poly(lactic acid)//cellulose acetate	0.3	0.000491	26
poly(lactic acid)//cellulose acetate	1.3	0.00075	51
poly(lactic acid)//chitin	0.017	0.44	11
poly(lactic acid)//chitin	0.029	0.49	12.5
poly(prop-1-ene)//poly(prop-1-ene)//poly(hexano-6-lactam)	0.048	0.001042	0
poly(prop-1-ene)//poly(prop-1-ene)//poly(hexano-6-lactam)	0.05	0.01688	0
poly(prop-1-ene)//polystyrene	0.25	0.005	0
poly(prop-1-ene)//polystyrene	0.21	0.0309	0
poly(prop-1-ene)//poly(methyl methacrylate)	0.84	0.034	4
poly(prop-1-ene)//poly(methyl methacrylate)	2.6	0.038	1000
poly(prop-1-ene)//poly(vinyl chloride)	0	0	14
poly(prop-1-ene)//poly(vinyl chloride)	0	0	23
poly(lactic acid)	0.26	0	1
poly(lactic acid)	0.66	0	2654
poly(ethylene oxide)//poly[(glycolic acid)-co-(lactic acid)]	0.0035	0	0.83
poly(ethylene oxide)//poly[(glycolic acid)-co-(lactic acid)]	2.17	0	1.93
polyethene//poly(prop-1-ene)	0.03	0	5
polyethene//poly(prop-1-ene)	0.13	0	950
poly(prop-1-ene)//poly(dodecano-12-lactam)	1.3	0	15
poly(prop-1-ene)//poly(dodecano-12-lactam)	1.7	0	23
poly(dimethylsiloxane)	0.00031	0	9
poly(dimethylsiloxane)	0.0076	0	637
poly(pentano-5-lactone)	0.57	0.57	150
poly(pentano-5-lactone)	0.57	0.57	200
Polyformaldehyde	1	0.028	20
Polyformaldehyde	3.6	0.097	380
poly(isobutylene oxide)	0.81	0.03103	235
poly(isobutylene oxide)	0.81	0.03103	235
poly[ethene-co-(vinyl acetate)]	0.0038	0.003	252.2
poly[ethene-co-(vinyl acetate)]	0.0062	0.0059	990
poly[ethene-co-(methyl acrylate)]	0.005	0	1140
poly[ethene-co-(methyl acrylate)]	0.005	0	1140
poly[(glycolic acid)-co-(lactic acid)]	1.3	0.043	5
poly[(glycolic acid)-co-(lactic acid)]	1.9	0.052	10.8
poly[(D-lactic acid)-co-(L-lactic acid)]	0.27	0.74	2.83
poly[(D-lactic acid)-co-(L-lactic acid)]	1.8	0.74	317
cellulose acetate	2	0	6
cellulose acetate	2	0	6
cellulose//amylopectin	0.56	0.011	8.4
cellulose//amylopectin	2.2	0.037	44
poly[ethene-co-(oct-1-ene)]//poly[ethene-co-(vinyl alcohol)]	0.035	0.0075	16
poly[ethene-co-(oct-1-ene)]//poly[ethene-co-(vinyl alcohol)]	1.1	0.0096	1025
poly(prop-1-ene)//poly(hexano-6-lactam)//poly[propylene-graft-(maleic anhydride)]	1.8	0.0383	5.3
poly(prop-1-ene)//poly(hexano-6-lactam)//poly[propylene-graft-(maleic anhydride)]	3.1	0.051	13.5
poly[ethylene-co-(but-1-ene)]//poly[ethene-co-(methyl acrylate)]	0.03	0.0055	101
poly[ethylene-co-(but-1-ene)]//poly[ethene-co-(methyl acrylate)]	0.11	0.0121	608
polyethene//polystyrene	0.2	0.0251	4.3
polyethene//polystyrene	3.3	0.0316	60
poly(lactic acid)//poly[ethene-co-(vinyl acetate)]	1.1	0.0285	1
poly(lactic acid)//poly[ethene-co-(vinyl acetate)]	1.8	0.0331	21.9
poly[ethene-co-(vinyl alcohol)]	0.88	0.055	40
poly[ethene-co-(vinyl alcohol)]	3.8	0.055	210

**Table 3:** The properties of articular cartilages (i.e., the target properties of this study).

Properties	Numerical Value	References
Elongation of facture	2 to 140%	[55]
Tensile strength	~35 MPa	[56,57]
Tensile Modulus	3 to 100 MPa	[55]

**Table 4:** Multinomial logistic regression goodness fit results.

S. No.	Model	Null_Residual Deviation	Model_Residual Deviation	Df	*p* Value
1	Tensile modulus	1763.898	1466.635	94	0
2	Tensile strength at yield	1763.898	1419.2	94	1.34 × 10^−9^
3	Elongation at break	1763.898	1564.195	94	0
4	Tensile modulus and elongation at break	1763.898	1075.018	94	0
5	Tensile strength and elongation at break	1763.898	1132.967	94	0
6	Tensile modulus and Tensile strength	1763.898	1188.942	94	0
7	Tensile modulus, tensile strength and elongation at break	1763.898	782.0324	94	0

**Table 5:** Predicted outputs (i.e., polymers/blends) from the multinomial logistic regression modeling.

Parameter	Properties	Prediction
Tensile modulus	Residual Deviance: 618.4709 AIC: 994.4709	(1) poly(glycolic acid)//poly(lactic acid) (2) poly(methyl methacrylate)//poly(epsilon-caprolactone)
Tensile strength at yield	Residual Deviance: 1410.82 AIC: 1786.82	(1) poly(lactic acid)
Elongation at break	Residual Deviance: 1460.5 AIC: 2212.5	(1) poly(lactic acid) (2) poly(prop-1-ene)//poly(ethylene terephthalate)//poly[ethene-co-(5-ethylidene-2-norbornene)-co-(prop-1-ene)]
Tensile modulus and elongation at break	Residual Deviance: 1186.365 AIC: 1750.365	(1) poly(vinyl chloride) (2) polyethene//polyethene-graft-poly(maleic anhydride)
Tensile strength and elongation at break	Residual Deviance: 1028.616 AIC: 1592.616	(1) poly(isobutylene oxide) (2) polyethene//polyethene-graft-poly(maleic anhydride)
Tensile modulus and Tensile strength	Residual Deviance: 675.875 AIC: 1239.875	(1) polyethene
Tensile modulus, tensile strength and elongation at break	Residual Deviance: 7.439214 AIC: 2212.5	(1) polyethene//polyethene-graft-poly(maleic anhydride)

## Data Availability

The data is included with the manuscript and the source code will be made available in Supplementary section.

## Supplementary Materials

The following supporting information can be downloaded at: https://www.mdpi.com/article/10.3390/polym14091802/s1. The basic code to carry multinomial logistic regression is attached.

## References

[1] Rathan S., Dejob L., Schipani R., Haffner B., Möbius M.E., Kelly D.J. (2019). Fiber Reinforced Cartilage ECM Functionalized Bioinks for Functional Cartilage Tissue Engineering. Adv. Healthc. Mater..

[2] Daly A.C., Freeman F.E., Gonzalez-Fernandez T., Critchley S.E., Nulty J., Kelly D.J. (2017). 3D Bioprinting for Cartilage and Osteochondral Tissue Engineering. Adv. Healthc. Mater..

[3] Collins A.T., Hu G., Newman H., Reinsvold M.H., Goldsmith M.R., Twomey-Kozak J.N., Leddy H.A., Sharma D., Shen L., DeFrate L.E. (2021). Obesity Alters the Collagen Organization and Mechanical Properties of Murine Cartilage. Sci. Rep..

[4] Roy H.S., Singh R., Ghosh D. (2021). Recent Advances in Nanotherapeutic Strategies That Target Nitric Oxide Pathway for Preventing Cartilage Degeneration. Nitric Oxide.

[5] Nishimura R., Hata K., Takahata Y., Murakami T., Nakamura E., Ohkawa M., Ruengsinpinya L. (2020). Role of Signal Transduction Pathways and Transcription Factors in Cartilage and Joint Diseases. Int. J. Mol. Sci..

[6] García-Martínez L., Campos F., Godoy-Guzmán C., del Carmen Sánchez-Quevedo M., Garzón I., Alaminos M., Campos A., Carriel V. (2017). Encapsulation of Human Elastic Cartilage-Derived Chondrocytes in Nanostructured Fibrin-Agarose Hydrogels. Histochem. Cell Biol..

[7] Anderson D.E., Johnstone B. (2017). Dynamic Mechanical Compression of Chondrocytes for Tissue Engineering: A Critical Review. Front. Bioeng. Biotechnol..

[8] Mandelbaum B.R., ElAttrache N.S. (2016). Articular Cartilage Repair Techniques. Sports Med. Arthrosc. Rev..

[9] Ashraf S., Cha B.-H., Kim J.-S., Ahn J., Han I., Park H., Lee S.-H. (2016). Regulation of Senescence Associated Signaling Mechanisms in Chondrocytes for Cartilage Tissue Regeneration. Osteoarthr. Cartil..

[10] Deng Z., Jin J., Zhao J., Xu H. (2016). Cartilage Defect Treatments: With or without Cells? Mesenchymal Stem Cells or Chondrocytes? Traditional or Matrix-Assisted? A Systematic Review and Meta-Analyses. Stem Cells Int..

[11] Hendren L., Beeson P. (2009). A Review of the Differences between Normal and Osteoarthritis Articular Cartilage in Human Knee and Ankle Joints. Foot Edinb. Scotl..

[12] Mourad A.-H.I., Akkad R.O., Soliman A.A., Madkour T.M. (2009). Characterisation of Thermally Treated and Untreated Polyethylene–Polypropylene Blends Using DSC, TGA and IR Techniques. Plast. Rubber Compos..

[13] Xu T., Miszuk J.M., Zhao Y., Sun H., Fong H. (2015). Electrospun Polycaprolactone 3D Nanofibrous Scaffold with Interconnected and Hierarchically Structured Pores for Bone Tissue Engineering. Adv. Healthc. Mater..

[14] Luca A.D., Lorenzo-Moldero I., Mota C., Lepedda A., Auhl D., Blitterswijk C.V., Moroni L. (2016). Tuning Cell Differentiation into a 3D Scaffold Presenting a Pore Shape Gradient for Osteochondral Regeneration. Adv. Healthc. Mater..

[15] Mourad A.-H.I., Abdel-Magid B.M., El-Maaddawy T., Grami M.E. (2010). Effect of Seawater and Warm Environment on Glass/Epoxy and Glass/Polyurethane Composites. Appl. Compos. Mater..

[16] Zhou Z., Wu W., Fang J., Yin J. (2020). Polymer-Based Porous Microcarriers as Cell Delivery Systems for Applications in Bone and Cartilage Tissue Engineering. Int. Mater. Rev..

[17] Abu-Jdayil B., Mourad A.-H.I., Hussain A. (2016). Investigation on the Mechanical Behavior of Polyester-Scrap Tire Composites. Constr. Build. Mater..

[18] Zhang Y., Liu X., Zeng L., Zhang J., Zuo J., Zou J., Ding J., Chen X. (2019). Polymer Fiber Scaffolds for Bone and Cartilage Tissue Engineering. Adv. Funct. Mater..

[19] Asadi N., Alizadeh E., Salehi R., Khalandi B., Davaran S., Akbarzadeh A. (2018). Nanocomposite Hydrogels for Cartilage Tissue Engineering: A Review. Artif. Cells Nanomed. Biotechnol..

[20] Attarian Shandiz M., Gauvin R. (2016). Application of Machine Learning Methods for the Prediction of Crystal System of Cathode Materials in Lithium-Ion Batteries. Comput. Mater. Sci..

[21] Chen C.-T., Gu G.X. (2020). Generative Deep Neural Networks for Inverse Materials Design Using Backpropagation and Active Learning. Adv. Sci..

[22] Silwattananusarn T., Tuamsuk K. (2012). Data Mining and Its Applications for Knowledge Management: A Literature Review from 2007 to 2012. arXiv.

[23] AbuOmar O., Nouranian S., King R., Bouvard J.L., Toghiani H., Lacy T.E., Pittman C.U. (2013). Data Mining and Knowledge Discovery in Materials Science and Engineering: A Polymer Nanocomposites Case Study. Adv. Eng. Inform..

[24] Mairpady A., Mourad A.-H.I., Mozumder M.S. (2021). Statistical and Machine Learning-Driven Optimization of Mechanical Properties in Designing Durable HDPE Nanobiocomposites. Polymers.

[25] Moot T., Isayev O., Call R.W., McCullough S.M., Zemaitis M., Lopez R., Cahoon J.F., Tropsha A. (2016). Material Informatics Driven Design and Experimental Validation of Lead Titanate as an Aqueous Solar Photocathode. Mater. Discov..

[26] Ramakrishna S., Zhang T.-Y., Lu W.-C., Qian Q., Low J.S.C., Yune J.H.R., Tan D.Z.L., Bressan S., Sanvito S., Kalidindi S.R. (2019). Materials Informatics. J. Intell. Manuf..

[27] Kajita S., Ohba N., Jinnouchi R., Asahi R. (2017). A Universal 3D Voxel Descriptor for Solid-State Material Informatics with Deep Convolutional Neural Networks. Sci. Rep..

[28] Singh A.V., Ansari M.H.D., Rosenkranz D., Maharjan R.S., Kriegel F.L., Gandhi K., Kanase A., Singh R., Laux P., Luch A. (2020). Artificial Intelligence and Machine Learning in Computational Nanotoxicology: Unlocking and Empowering Nanomedicine. Adv. Healthc. Mater..

[29] Zhang K., Wang J., Liu T., Luo Y., Loh X.J., Chen X. (2021). Machine Learning-Reinforced Noninvasive Biosensors for Healthcare. Adv. Healthc. Mater..

[30] Kim K., Kang S., Yoo J., Kwon Y., Nam Y., Lee D., Kim I., Choi Y.-S., Jung Y., Kim S. (2018). Deep-Learning-Based Inverse Design Model for Intelligent Discovery of Organic Molecules. Npj Comput. Mater..

[31] Zunger A. (2018). Inverse Design in Search of Materials with Target Functionalities. Nat. Rev. Chem..

[32] Venkatraman V., Alsberg B.K. (2018). Designing High-Refractive Index Polymers Using Materials Informatics. Polymers.

[33] Tao L., Varshney V., Li Y. (2021). Benchmarking Machine Learning Models for Polymer Informatics: An Example of Glass Transition Temperature. J. Chem. Inf. Model..

[34] Ishikiriyama K. (2022). Polymer Informatics Based on the Quantitative Structure-Property Relationship Using a Machine-Learning Framework for the Physical Properties of Polymers in the ATHAS Data Bank. Thermochim. Acta.

[35] Chen L., Kern J., Lightstone J.P., Ramprasad R. (2021). Data-Assisted Polymer Retrosynthesis Planning. Appl. Phys. Rev..

[36] Le T.-T. (2020). Prediction of Tensile Strength of Polymer Carbon Nanotube Composites Using Practical Machine Learning Method. J. Compos. Mater..

[37] Kim B., Lee S., Kim J. (2020). Inverse Design of Porous Materials Using Artificial Neural Networks. Sci. Adv..

[38] Garcovich D., Marques Martinez L., Adobes Martin M. (2020). Citation Classics in Paediatric Dentistry: A Bibliometric Study on the 100 Most-Cited Articles. Eur. Arch. Paediatr. Dent..

[39] Yu D., He X. (2020). A Bibliometric Study for DEA Applied to Energy Efficiency: Trends and Future Challenges. Appl. Energy.

[40] Goldring M.B., Goldring S.R. (2007). Osteoarthritis. J. Cell. Physiol..

[41] Muzzarelli R.A.A. (2009). Chitins and Chitosans for the Repair of Wounded Skin, Nerve, Cartilage and Bone. Carbohydr. Polym..

[42] Puppi D., Chiellini F., Piras A.M., Chiellini E. (2010). Polymeric Materials for Bone and Cartilage Repair. Prog. Polym. Sci..

[43] Huey D.J., Hu J.C., Athanasiou K.A. (2012). Unlike Bone, Cartilage Regeneration Remains Elusive. Science.

[44] Chung C., Burdick J.A. (2008). Engineering Cartilage Tissue. Adv. Drug Deliv. Rev..

[45] Lee S.-H., Shin H. (2007). Matrices and Scaffolds for Delivery of Bioactive Molecules in Bone and Cartilage Tissue Engineering. Adv. Drug Deliv. Rev..

[46] Makris E.A., Gomoll A.H., Malizos K.N., Hu J.C., Athanasiou K.A. (2015). Repair and Tissue Engineering Techniques for Articular Cartilage. Nat. Rev. Rheumatol..

[47] Liu M., Zeng X., Ma C., Yi H., Ali Z., Mou X., Li S., Deng Y., He N. (2017). Injectable Hydrogels for Cartilage and Bone Tissue Engineering. Bone Res..

[48] Vinatier C., Mrugala D., Jorgensen C., Guicheux J., Noël D. (2009). Cartilage Engineering: A Crucial Combination of Cells, Biomaterials and Biofactors. Trends Biotechnol..

[49] Balakrishnan B., Banerjee R. (2011). Biopolymer-Based Hydrogels for Cartilage Tissue Engineering. Chem. Rev..

[50] Teoh S.H. (2000). Fatigue of Biomaterials: A Review. Int. J. Fatigue.

[51] Qu H., Fu H., Han Z., Sun Y. (2019). Biomaterials for Bone Tissue Engineering Scaffolds: A Review. RSC Adv..

[52] Kerin A.J., Wisnom M.R., Adams M.A. (1998). The Compressive Strength of Articular Cartilage. Proc. Inst. Mech. Eng..

[53] Sophia Fox A.J., Bedi A., Rodeo S.A. (2009). The Basic Science of Articular Cartilage: Structure, Composition, and Function. Sports Health.

[54] Langworthy M.J., Nelson F.R.T., Coutts R.D. (2004). Basic Science. Articul. Cartil. Lesions.

[55] Nordin M., Frankel V.H. (2001). Basic Biomechanics of the Musculoskeletal System.

[56] Lu X.L., Mow V.C. (2008). Biomechanics of Articular Cartilage and Determination of Material Properties. Med. Sci. Sports Exerc..

[57] Bellucci G. (2001). Mechanical Behaviour of Articular Cartilage under Tensile Cyclic Load. Rheumatology.

[58] Otsuka S., Kuwajima I., Hosoya J., Xu Y., Yamazaki M. PoLyInfo: Polymer Database for Polymeric Materials Design. Proceedings of the 2011 International Conference on Emerging Intelligent Data and Web Technologies.

[59] Jeune W., Francelino M., de Souza E., Fernandes-Filho E., Rocha G. (2018). Multinomial Logistic Regression and Random Forest Classifiers in Digital Mapping of Soil Classes in Western Haiti. Rev. Bras. Ciênc. Solo.

[60] Hilbe J.M. (2009). Logistic Regression Models.

[61] Itano K., Ueki K., Iizuka T., Kuwatani T. (2020). Geochemical Discrimination of Monazite Source Rock Based on Machine Learning Techniques and Multinomial Logistic Regression Analysis. Geosciences.

[62] Sweileh W.M. (2020). Bibliometric Analysis of Scientific Publications on “Sustainable Development Goals” with Emphasis on “Good Health and Well-Being” Goal (2015–2019). Glob. Health.

[63] Yue T., Liu H., Long R., Chen H., Gan X., Liu J. (2020). Research Trends and Hotspots Related to Global Carbon Footprint Based on Bibliometric Analysis: 2007–2018. Environ. Sci. Pollut. Res..

[64] Vinatier C., Bouffi C., Merceron C., Gordeladze J., Brondello J.-M., Jorgensen C., Weiss P., Guicheux J., Noël D. (2009). Cartilage Tissue Engineering: Towards a Biomaterial-Assisted Mesenchymal Stem Cell Therapy. Curr. Stem Cell Res. Ther..

[65] Merceron C., Portron S., Masson M., Fellah B.H., Gauthier O., Lesoeur J., Chérel Y., Weiss P., Guicheux J., Vinatier C. (2010). Cartilage Tissue Engineering: From Hydrogel to Mesenchymal Stem Cells. Biomed. Mater. Eng..

[66] van Osch G.J.V.M., Brittberg M., Dennis J.E., Bastiaansen-Jenniskens Y.M., Erben R.G., Konttinen Y.T., Luyten F.P. (2009). Cartilage Repair: Past and Future—Lessons for Regenerative Medicine. J. Cell. Mol. Med..

[67] Aria M., Cuccurullo C. (2017). Bibliometrix: An R-Tool for Comprehensive Science Mapping Analysis. J. Informetr..

[68] Callaghan J.J. (2003). The Adult Knee.

[69] Hatton J., Davis G.R., Mourad A.-H.I., Cherupurakal N., Hill R.G., Mohsin S. (2019). Fabrication of Porous Bone Scaffolds Using Alginate and Bioactive Glass. J. Funct. Biomater..

[70] Petrucci C.J. (2009). A Primer for Social Worker Researchers on How to Conduct a Multinomial Logistic Regression. J. Soc. Serv. Res..

[71] Böhning D. (1992). Multinomial Logistic Regression Algorithm. Ann. Inst. Stat. Math..

[72] McCaffrey B.J. (2016). Neural Networks Using the R Nnet Package. https://visualstudiomagazine.com/articles/2016/11/01/using-the-r-nnet-package.aspx.

[73] Perego G., Cella G.D., Bastioli C. (1996). Effect of Molecular Weight and Crystallinity on Poly(Lactic Acid) Mechanical Properties. J. Appl. Polym. Sci..

[74] Deshmukh K., Basheer Ahamed M., Deshmukh R.R., Khadheer Pasha S.K., Bhagat P.R., Chidambaram K., Sadasivuni K.K., Ponnamma D., Kim J., Cabibihan J.-J., AlMaadeed M.A. (2017). 3-Biopolymer Composites With High Dielectric Performance: Interface Engineering. Biopolymer Composites in Electronics.

[75] Ansari M.M., Ahmad A., Kumar A., Alam P., Khan T.H., Jayamurugan G., Raza S.S., Khan R. (2021). Aminocellulose-Grafted-Polycaprolactone Coated Gelatin Nanoparticles Alleviate Inflammation in Rheumatoid Arthritis: A Combinational Therapeutic Approach. Carbohydr. Polym..

[76] Baharlou Houreh A., Masaeli E., Nasr-Esfahani M.H. (2021). Chitosan/Polycaprolactone Multilayer Hydrogel: A Sustained Kartogenin Delivery Model for Cartilage Regeneration. Int. J. Biol. Macromol..

[77] Augustine R., Dan P., Schlachet I., Rouxel D., Menu P., Sosnik A. (2019). Chitosan Ascorbate Hydrogel Improves Water Uptake Capacity and Cell Adhesion of Electrospun Poly(Epsilon-Caprolactone) Membranes. Int. J. Pharm..

[78] Eschbach F.O., Huang S.J. (1994). Hydrophilic-Hydrophobic Binary Systems of Poly(2-Hydroxyethyl Methacrylate) and Polycaprolactone. Part I: Synthesis and Characterization. J. Bioact. Compat. Polym..

[79] Tannoury C., Oguz E., Stock G.H., Brown A.K., Anderson D.G., Vaccaro A.R., Haid R.W., Papadopoulos S., Sasso R.C., Traynelis V.C. (2007). CHAPTER 9-Biomaterials in Spinal Arthroplasty. Spinal Arthroplasty.

[80] Hacker M.C., Krieghoff J., Mikos A.G., Atala A., Lanza R., Mikos A.G., Nerem R. (2019). Chapter 33-Synthetic Polymers. Principles of Regenerative Medicine.

[81] Mazzoccoli J.P., Feke D.L., Baskaran H., Pintauro P.N. (2010). Mechanical and Cell Viability Properties of Crosslinked Low- and High-Molecular Weight Poly(Ethylene Glycol) Diacrylate Blends. J. Biomed. Mater. Res. A.

[82] Patrício T., Bártolo P. (2013). Thermal Stability of PCL/PLA Blends Produced by Physical Blending Process. Procedia Eng..

[83] Baptista R., Guedes M. (2021). Morphological and Mechanical Characterization of 3D Printed PLA Scaffolds with Controlled Porosity for Trabecular Bone Tissue Replacement. Mater. Sci. Eng. C.

[84] Weijie Z., Zhuo C., Sujuan M., Yonggang W., Fei Z., Keyi W., Chenguang Y., Xiuying P., Jianzhong M., Yuli W. (2016). Cistanche Polysaccharide (CDPS)/Polylactic Acid (PLA) Scaffolds Based Coaxial Electrospinning for Vascular Tissue Engineering. Int. J. Polym. Mater. Polym. Biomater..

[85] Mourad A.-H., Mozumder M., Mairpady A., Pervez H., Kannuri U. (2017). On the Injection Molding Processing Parameters of HDPE-TiO_2_ Nanocomposites. Materials.

[86] Mozumder M.S., Mairpady A., Mourad A.-H.I. (2019). HDPE/TiO_2_ Nanocomposite: Fabrication and Optimization of Mechanical Property by RSM and ANN. Solid State Phenom..

[87] Mozumder M.S., Mourad A.-H.I., Mairpady A., Pervez H., Haque M.E. (2018). Effect of TiO_2_ Nanofiller Concentration on the Mechanical, Thermal and Biological Properties of HDPE/TiO_2_ Nanocomposites. J. Mater. Eng. Perform..

[88] Mozumder M.S., Mairpady A., Mourad A.-H.I. (2017). Polymeric Nanobiocomposites for Biomedical Applications. J. Biomed. Mater. Res. B Appl. Biomater..

[89] Remya N.S., Nair P.D. (2013). Engineering Cartilage Tissue Interfaces Using a Natural Glycosaminoglycan Hydrogel Matrix—An in Vitro Study. Mater. Sci. Eng. C.

[90] Ciorba A., Martini A. (2006). Tissue Engineering and Cartilage Regeneration for Auricular Reconstruction. Int. J. Pediatr. Otorhinolaryngol..

[91] Barkhad M.S., Abu-Jdayil B., Mourad A.H.I., Iqbal M.Z. (2020). Thermal Insulation and Mechanical Properties of Polylactic Acid (PLA) at Different Processing Conditions. Polymers.

[92] Bernardes G.P., da Luiz N.R., Santana R.M.C., de Camargo Forte M.M. (2020). Influence of the Morphology and Viscoelasticity on the Thermomechanical Properties of Poly(Lactic Acid)/Thermoplastic Polyurethane Blends Compatibilized with Ethylene-Ester Copolymer. J. Appl. Polym. Sci..

[93] Kaavessina M., Chafidz A., Ali I., Al-Zahrani S.M. (2015). Characterization of Poly(Lactic Acid)/Hydroxyapatite Prepared by a Solvent-Blending Technique: Viscoelasticity and in Vitro Hydrolytic Degradation. J. Elastomers Plast..

[94] Shahin-Shamsabadi A., Hashemi A., Tahriri M., Bastami F., Salehi M., Mashhadi Abbas F. (2018). Mechanical, Material, and Biological Study of a PCL/Bioactive Glass Bone Scaffold: Importance of Viscoelasticity. Mater. Sci. Eng. C.

[95] Sundgren N., Bergman G., Shur Y.J. (1978). Antiplasticization and Transition to Marked Nonlinear Viscoelasticity in Poly(Vinyl Chloride) (PVC)/Poly-ε-Caprolactone (PCL) Blends. J. Appl. Polym. Sci..

[96] Izuka A., Winter H.H., Hashimoto T. (1992). Molecular Weight Dependence of Viscoelasticity of Polycaprolactone Critical Gels. Macromolecules.

[97] Ionita S., Popescu S., Lascar I. (2015). Polypropylene Meshes and Other Alloplastic Implants for Soft Tissue and Cartilage Nasal Reconstructive Surgery—A Literature Review. Romanian J. Rhinol..

[98] Li X., Chen S., Li J., Wang X., Zhang J., Kawazoe N., Chen G. (2016). 3D Culture of Chondrocytes in Gelatin Hydrogels with Different Stiffness. Polymers.

[99] Erickson I.E., van Veen S.C., Sengupta S., Kestle S.R., Mauck R.L. (2011). Cartilage Matrix Formation by Bovine Mesenchymal Stem Cells in Three-Dimensional Culture Is Age-Dependent. Clin. Orthop..

