# Supplementary material for: Accelerated Discovery of the Polymer Blends for Cartilage Repair through Data-Mining Tools and Machine-Learning Algorithm
Source: Polymers (Basel). 2022 Apr 28;14(9):1802. doi: 10.3390/polym14091802 (PMC9104973; doi:10.3390/polym14091802)
Supplement: Supplementary file 1 [file polymers-14-01802-s001.zip › polymers-1672795-supplementary.pdf]

Programming Code:

```
install.packages(‘nnet’)

library(‘nnet’)

# Read the file in the .csv format

data.polymer <-read.csv(“database.csv)

# Read the numerical variable in numerical format

data.polymer$input1 <- as.numeric (data.polymer$input1)

data.polymer$input2 <- as.numeric (data.polymer$input2)

data.polymer$input3 <- as.numeric (data.polymer$input3)

data.polymer$output1 <- as.numeric (data.polymer$output1)

# Read the categorical variable in numerical format

data.polymer$input1 <- as.numeric (data.polymer$input1)

data.polymer$input2 <- as.numeric (data.polymer$input2)

data.polymer$input3 <- as.numeric (data.polymer$input3)

data.polymer$output1 <- as.numeric (data.polymer$output1)

# Incase the input has any missing value identify and take of the overall average value

data.polymer$Input1 = ifelse(is.na(data.polymer$Input1),

                ave(data.polymer$Input1, FUN = function(x) mean(x, na.rm = TRUE)),

                data.polymer$Input1)

data.polymer$Input2 = ifelse(is.na(data.polymer$Input2),

                ave(data.polymer$Input2, FUN = function(x) mean(x, na.rm = TRUE)),

                data.polymer$Input2)

data.polymer$Input3 = ifelse(is.na(data.polymer$ Input3),

               ave(data.polymer$Input3, FUN = function(x) mean(x, na.rm = TRUE)),

               data.polymer$Input3)

# Incase the value between the three factors have variation there need to scale data

data.polymer[-1] <- scale(data.polymer[-1])

# Modelling of data using multinomial logistic regression

model <- multinom(Polymer ~ INPUT2+E+INPUT1, data=data.polymer)
```

```
# Checking for the models goodness of fit

multimodelfit1 <- multinom (data = data.polymer,

                           Polymer ~ 1)

multimodelfit2 <- multinom (data = data.polymer,

                            Polymer ~ variable)

anova(multimodelfit1, multimodelfit2)

# Predict the model inserting programmer desired input

 y_pred = predict(model, data.frame(input1= range(seq(min:max)), input2 =range(seq(min:max)), input3
= (min:max))
```
